# A Medical Mosaic—The Composite Board of Medical Examiners

**Published:** 1907-09

**Authors:** 


### EDITORIAL DEPARTMENT.

### A MEDICAL MOSAIC—THE COMPOSITE BOARD OF MEDICAL EXAMINERS.

The State Medical Association is fairly launched on an untried sea. It has made a radical departure from its record of forty years, has gone back on its declared principles and policy, and has inaugurated the "new dispensation" with a vengeance. Twenty years ago it would expel a member for affiliating with a homo on a board (see Transactions 1877), and in 1896 it passed a resolution declaring that "We will expel any member who will consult with a homeopath." All the pathies, especially in later years, the osteos, have come in for sneers, denunciation and abuse; but "times change and men change with them." The year of jubilee seems to have come, indeed, "and the lion and the lamb lie down together." To see two honored ex-Presidents of the State Medical Association serving on an examining board with an osteopath is a sight to make the shades of Swearingen, Ashbel Smith, Cupples, Starley and Becton go weep,—if a shade can weep.

The new board goes to work under the presidency of Dr. M. E. Daniel, eclectic, of Honey Grove; the vice-presidency of Dr. Crowe, a homeopath; and ex-President of the State Medical Association, Dr. Foscue, to whose management and influence more than any other is due the passage and signing of this crazy-quilt bill, is Secretary-Treasurer.

The State, by authority of the Constitution, undertakes to regulate the practice of medicine and fix a standard of qualification for physicians. To this end it requires an examination in everything *except the essentials*, the practice of medicine and materia medica and therapeutics. What a paradox! What a farce!

But it is the law, and must be obeyed. I see breakers ahead, and predict a repeal of the act next session. The Assistant Attorney General has twice tried his hand at construing it, but it can't be construed. I give below his special construction of certain parts, at request of the board. He has unraveled some of the kinks, it is true, but it is verily a knotty problem.

The Governor announced the appointment of the board the day after the August JOURNAL was mailed. It consists of five regulars, two eclectics, two homeopaths, one physio-medic and one osteopath.

"No school shall have a majority on the board," but by concert and agreement the four "minor schools" can control its action and elect the officers from their ranks,—as witness the election of its first president and vice-president, while a representative of the dominant school,—of an Association numbering 3300, or three-fourths of all the doctors in Texas, and its last President at that—must play second fiddle—and do all the work.

PERSONNEL OF THE BOARD.—*Regulars, State Medical Association*—Dr. G. B. Foscue, late President, Waco; Dr. Jas. D. Osborn, late President, Cleburne; Dr. E. P. Becton, Jr., Greenville; Dr. W. B. Collins, Lovelady; Dr. J. J. Dial, Sulphur Springs.

*Homeopaths*—Dr. J. D. Mitchell, Fort Worth; Dr. J. T. Crowe, Dallas.

*Eclectics*—Dr. M. E. Daniel, Honey Grove; Dr. J. P. Rice, Fredericksburg.

*Physio-Medical*—Dr. R. O. Braswell, Mineral Wells.

*Osteopath*—Dr. R. W. Collins, El Paso.

The Board met in Austin August 15th (ult.), organized, elected officers, asked for an opinion of the Attorney General, and apportion the work as below given:

President of the Board—Dr. M. E. Daniel, Honey Grove.

Vice-President—Dr. J. T. Crowe, Dallas.

Secretary-Treasurer—Dr. G. B. Foscue, Waco.

The following committees were appointed:

Rules and Regulations—T. J. Crowe, J. D. Osborn and R. O. Braswell.

Reciprocity—G. B. Foscue, E. P. Becton aand J. P. Rice.

Committee on Institutions to Make a Study of the Various Medical Colleges—W. B. Collins, J. P. Rice, J. D. Mitchell, R. O. Braswell and R. W. Collins.

Committee to Draft Certificates to Be Used—E. P. Becton, J. D. Mitchell and R. O. Braswell.

The different branches for examination were distributed as follows:

J. J. Dial, pathology and histology; T. J. Crowe, chemistry; J. D. Mitchell, obstetrics; G. B. Foscue, physical diagnosis; W. B.

Collins, anatomy; J. P. Rice, bacteriology; E. P. Becton, surgery; R. O. Braswell, gynecology; R. W. Collins, hygiene; M. E. Daniels, physiology.

Judge Pollard, Assistant Attorney General, at request of the board, submitted the following (this is not the "opinion" given of the act in its entirety, which opinion we did not publish because it came too late for July and too early for August; it had appeared elsewhere):

We are in receipt of yours of the 15th, submitting the following questions for our decision, viz.:

"First—Please advise us as to the eligibility on this board of osteopaths. (See Section 1, Board Medical Bill.)

"Second—Would these men who qualified under previous laws by the registration of diplomas and then afterwards obtained licenses from the three State boards, or any of the three State boards, be required to get a verification license under the existing law? Also render a complete construction of Section 6 as a whole.

"Third—Please render an opinion on the construction of the last four lines of Section 10.

"Fourth—Can Section 13 be so construed as to prohibit counter-prescribing by druggists and pharmacists?"

You are advised as to the first question:

Section 1 of the medical act provides that the Board of Medical Examiners shall consist of eleven men learned in medicine, legal and active practitioners in the State of Texas, who shall have resided and practiced medicine in this State under a diploma from a legal and reputable college of medicine of the school to which said practitioner shall belong for more than three years prior to their appointment.

An answer to your inquiry depends upon what is meant by "medicine" and "practice medicine" and "college of medicine" as used in Section 1.

Section 1 contains also the following provision: "The word 'medicine' as used in this section shall have the same meaning and scope as given to it in Section 13 of this act."

Section 13 provides that persons shall be regarded as practicing medicine within the meaning of this act "who shall treat or offer to treat any disease or disorder, mental or physical, or any physical deformity or injury by any system or method, or to effect a cure thereof, and charge therefor, directly or indirectly, money or other compensation."

I understand that this definition of "practicing medicine" includes within its terms that class of practitioners known as osteopaths. Being included within the definition of the term they are eligible to be appointed upon the Board of Medical Examiners.

Replying to your second question, you are advised that those practitioners who secured from either of the State medical examining boards, under the Act of 1901, a State license upon a diploma which was registered prior to January 1, 1891, are not required to secure verification license from the new board. Neither are these practitioners who secured license from one of the State medical boards, under the Act of 1901, upon a diploma which was registered after July 9, 1901, required to get a verification license.

Those practitioners who secured license from either of the State medical examining boards, under the Act of 1901, upon a diploma which was registered between January 1, 1891, and July 9, 1901, are required to present to the new board documents or legally certified transcripts of documents sufficient to establish the existence and validity of such diploma and the proper record thereof, and in addition thereto are required to satisfy the board that the diploma was issued from a bona fide medical college of reputable standing, and receive from the board a verification license.

It is only this class of practitioners, viz., those whose license from one of the State medical examining boards under the Act of 1901 was issued upon a diploma recorded between January 1, 1891, and July 9, 1901, that are required to secure a verification license under the new law. Those having a certificate from one of the State boards under the Act of 1901, which is not based upon a diploma registered between the dates January 1, 1891, and July 9, 1901, and those who secured such State license upon examination, or upon reciprocity, are not required to secure verification license, with this exception, that if the reciprocity license was based upon a diploma recorded between the dates, they will be required to present to the new board the documents establishing validity and record of diploma and, in addition, evidence that same was issued by a college of reputable standing, and secure verification license.

Further endeavoring to make my construction of the law clear, beg to advise as follows:

First—Those persons who were practicing medicine prior to 1885, if their right to practice depends entirely upon the fact that they were practicing prior to that date, are required to present to the new board evidence to establish the fact that they were practicing prior to this date. This may be done by affidavits, as the board may direct. A verification license must be issued to all this class.

Second—Those whose right to practice depends upon diplomas recorded from January 1, 1885, to January 1, 1891, are required to present to the new board evidence satisfactory to establish the existence and validity of such diplomas and proper record thereof, and receive from the board verification license.

Third—Those whose right to practice depends upon diplomas recorded between January 1, 1891, and July 9, 1901, are required to present to the new board evidence sufficient to establish the existence and validity of such diplomas and the proper record thereof and, in addition, satisfactory evidence that such diplomas were issued from bona fide medical colleges of reputable standing. This must be done regardless of the fact that such practitioners may have secured from one of the State boards, under the Act of 1901, a license, if such license was issued upon such diploma.

Fourth—Those whose right to practice depends upon certificates issued by district boards between January 1, 1885, and July 9, 1901, are required to present to the new board evidence sufficient to establish the existence and validity of such certificates and the proper record thereof, and receive from the new board verification license.

Fifth—Those whose right to practice is based upon an examination before one of the boards, under the Act of 1901, are not required to secure verification license; neither are those whose certificates are based upon reciprocity, unless such reciprocity had for its basis a diploma recorded between January 1, 1891, and July 9, 1901.

Replying to your third question, you are advised that the last four lines of Section 10 make it unlawful for persons to sell on the streets, or other public places, remedies which they recommend for the cure of diseases, unless such persons so selling are either licensed druggists or physicians qualified to practice medicine under the provisions of this act.

Replying to your fourth question, you are advised that a druggist can not treat any disease or disorder by prescription or otherwise, unless he has a license to practice medicine under the provisions of this act.

